# Isolated external iliac artery aneurysm in a patient without any concomitant vascular disease

**DOI:** 10.1016/j.jvscit.2024.101682

**Published:** 2024-11-16

**Authors:** Patrick D. Conroy, Jennifer Li, Allen Hamdan

**Affiliations:** a Department of Vascular and Endovascular Surgery, Beth Israel Deaconess Hospital, Boston, MA; b Department of Vascular and Endovascular Surgery, Cooper University Hospital, Camden, NJ

**Keywords:** Aneurysms, External iliac artery aneurysm, Isolated iliac artery aneurysm, Aortoiliac

## Abstract

Iliac artery aneurysms commonly present in patients with associated aortic disease. Isolated aneurysms of the iliac arteries are uncommon, mostly involving the common iliac artery. Isolated external iliac artery aneurysms are the rarest iliac aneurysmal pathology. We present the case of an asymptomatic isolated external iliac artery aneurysm with no other concomitant vascular disease in a middle-aged man, treated with a stent graft.

Isolated aneurysms of the iliac artery system are rare, with most reports being presented in case reports.^1^, ^2^, ^3^, ^4^ Based on autopsy data, it is estimated that the incidence of isolated iliac artery aneurysms is 0.03%.^5^ Even more rare, external iliac artery (EIA) aneurysms comprise <1% of all isolated iliac artery aneurysms.^6^ In this report, we describe a case of a man who had an incidental finding of an isolated EIA with no symptoms. After further investigation by the vascular surgery team, he had no other aortic or peripheral vascular disease and no personal or family history of vascular abnormalities. The patient provided written informed consent for the report of his case and imaging studies.

## Case report

A 68-year-old man was referred to the vascular surgery department for evaluation of an EIA aneurysm incidentally found on a screening magnetic resonance imaging after an elevated prostate-specific antigen on routine screening laboratory tests. Medical history was significant for type 2 diabetes mellitus, hypertension, hyperlipidemia, and coronary artery disease without a history of myocardial infarction or preventative intervention. He presented to the office and was evaluated for symptoms; he reported none. There was no history of trauma, infectious etiology, or prior vascular access. The patient denied any history of smoking, cycling or extreme sporting, or family history of aneurysms. Blood cultures were negative, and leukocytes were within normal limits.

**Fig 1:**
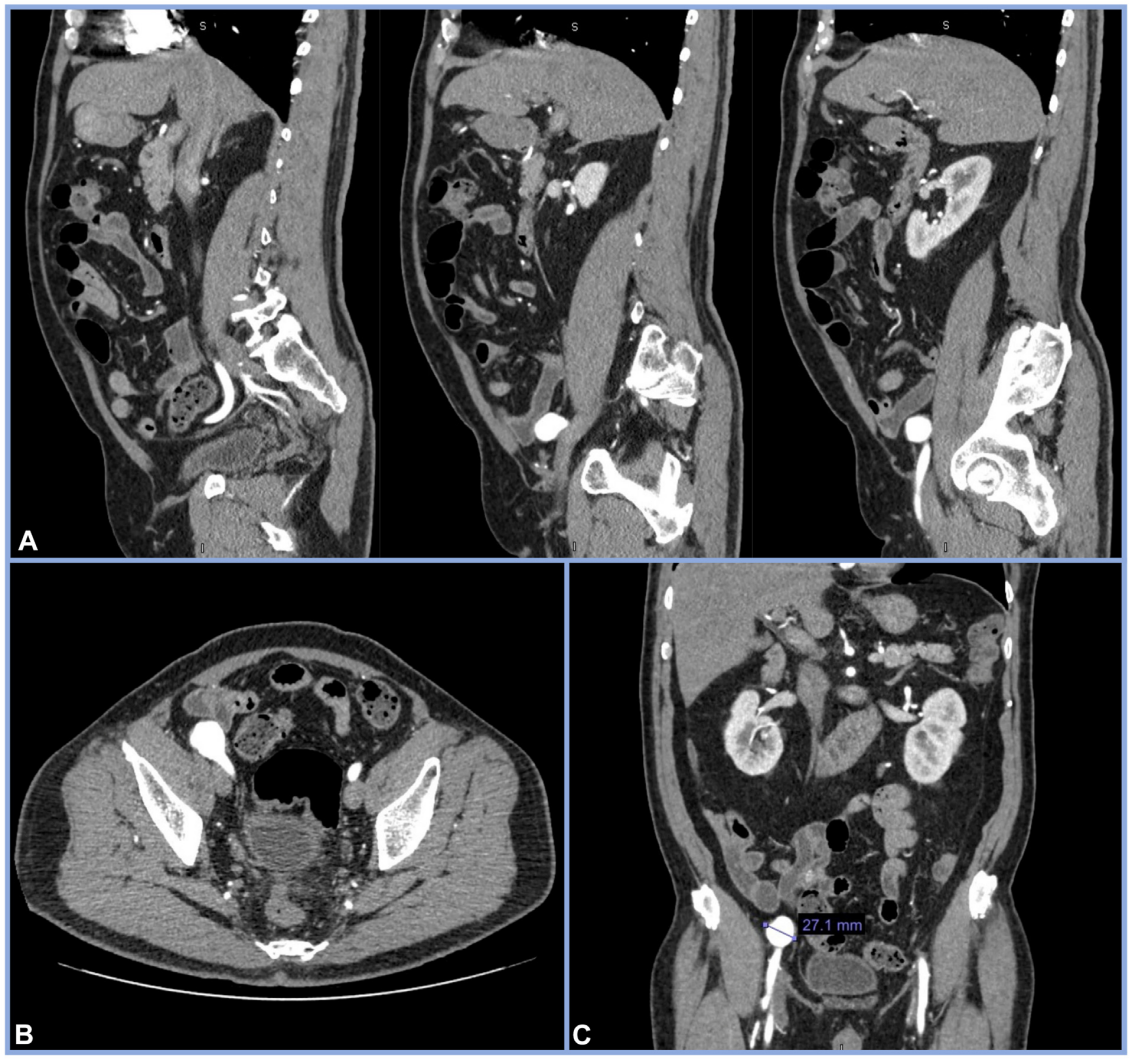
Preoperative computed tomography angiography showing an isolated aneurysm of the suprainguinal right external iliac artery (*EIA*). (**A**) Sagittal. (**B**) Axial. (**C**) Coronal with measurement.

Computed tomography angiogram of the chest, abdomen, and pelvis with runoff revealed an isolated right sided saccular 2.6-cm EIA aneurysm above the inguinal ligament, with no extension proximally or distally (Fig 1, *A*-*C*). There was no aneurysmal or major atherosclerotic disease in the abdominal aorta or distal arterial vessels and there were no signs of disease in the contralateral iliac arteries. Based on discussion with the vascular surgery team, the patient was given the options of endovascular vs open surgery. Using shared decision-making, an endovascular approach was chosen to treat the isolated EIA aneurysm, and the patient provided consent.

### Operative procedure

**Fig 2:**
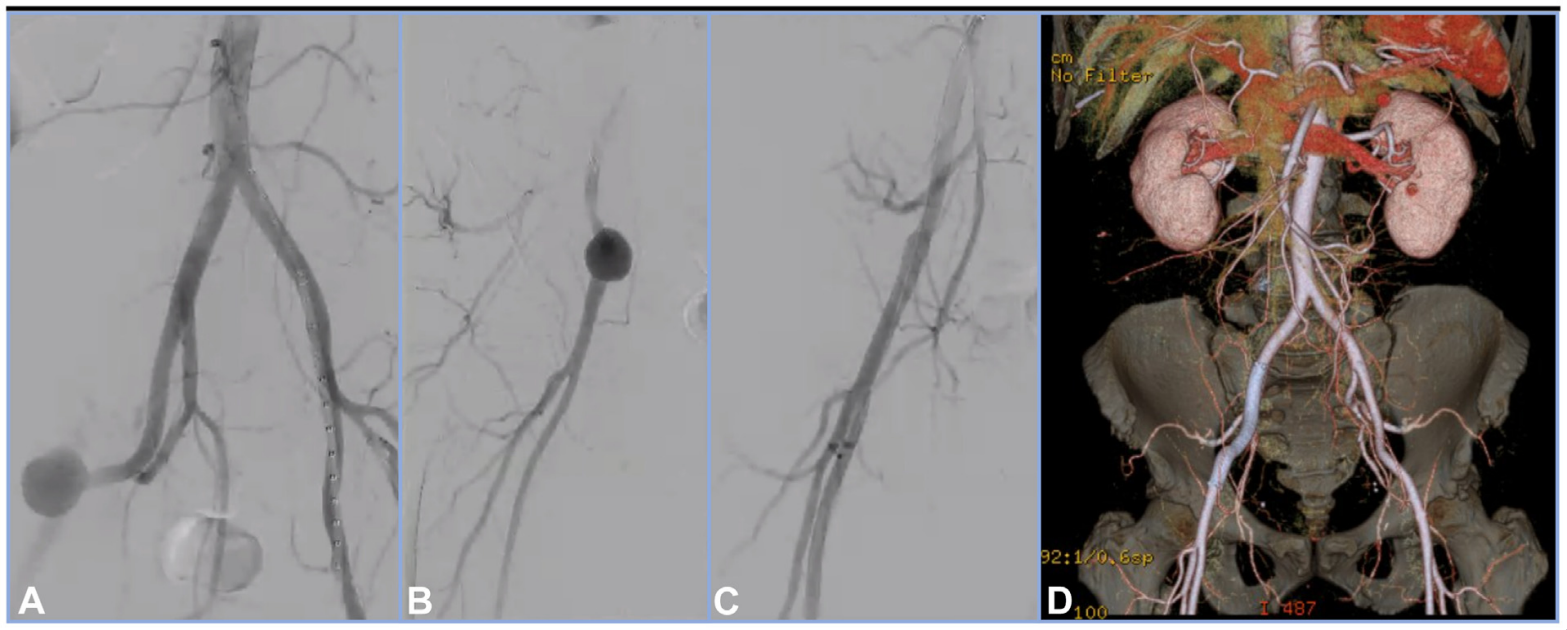
Intraoperative angiography. (**A**) Preoperative aortogram. (**B**) Preoperative iliac angiogram. (**C**) Completion angiogram after stent graft deployment. (**D**) Reconstructed three-dimensional image of 2-week follow-up computed tomography scan showing completely excluded aneurysm with patent stent graft in suprainguinal right external iliac artery (*EIA*).

The operation was performed with the patient under general anesthesia. An 8F short 25 cm sheath (Terumo Medical Co, Tokyo, Japan) was placed percutaneously at the left femoral artery, and the Omni Flush Soft-Vu Angiographic Catheter (Angiodynamics, Latham, NY) was advanced and positioned into the infrarenal abdominal aorta. An aortogram was captured (Fig 2, *A* and *B*) to visualize the iliac arteries with an oblique view to visualize the right hypogastric takeoff. A widely patent bilateral iliac system was visualized, and a large 2.6-m aneurysm was identified 4 cm above the femoral bifurcation. The Omni Flush over a floppy Glidewire (Terumo Medical Corp., Somerset, NJ) was advanced across the iliac bifurcation, beyond the aneurysm sac, and further down into the right superficial femoral artery. The short 8F sheath was exchanged for an 8F 45 cm Ansel Sheath (Cook Medical, Bloomington, IN) over a J-tipped Stiff Amplatz Wire (Boston Scientific, Natick, MA), and advanced to the mid-right EIA just proximal to the aneurysm. After heparinizing the patient and measuring the native vessel for optimal graft selection, the 9 mm × 10 cm Viabahn stent graft (W. L. Gore & Associates, Flagstaff, AZ) was ultimately selected and carefully deployed in a distal to proximal fashion. The entirety of the aneurysmal sac was covered while ensuring maintaining patency of the common femoral artery distally and hypogastric artery proximally. Final angiography (Fig 2, *C*) demonstrated widely patent right EIA and widely patent femoral bifurcation with complete exclusion of the EIA aneurysm. Given that the stent graft appeared to have an excellent seal, it was decided not to post-dilate with balloon angioplasty.

### Postoperative course

The patient was discharged home on postoperative day 1 without complication on a regimen of aspirin only, indefinitely. On outpatient review at 2 weeks, the patient was well. No difference in peripheral pulse examination was found. The patient was asymptomatic preoperatively and remained symptom free at the 2-week follow-up. Postoperative computed tomography angiography demonstrated an excluded aneurysm sac with a good apposition of the stent graft with no evidence of endoleaks or stent graft-related complications (Fig 2, *D*). We plan to perform annual ultrasound surveillance of the stent graft.

## Discussion

Common and internal iliac artery aneurysms have multifactorial pathogeneses that are nearly identical to that of abdominal aortic aneurysms, as seen by their histological similarities. The particular rarity of aneurysms involving the EIA can be attributed to the unique lamellar architecture and biomechanical properties of the external iliac arterial walls, particularly in the tunica media.^7^ Distinct from the more proximal aortoiliac segments, external iliac arteries possess a more structured and layered lamellar architecture, as well as a higher elastin-to-collagen ratio,^8^ which allows them to withstand higher hemodynamic stresses and accommodate higher pressures, thus reducing the susceptibility to wall weakening and aneurysmal dilation.

Isolated iliac artery aneurysms, without any other identifiable aortoiliac or peripheral vascular disease, have been described in multiple investigations to be a rare pathology. Silver et al^9^ in 1967 performed a chart review of patients with arterial aneurysms affecting the aortic or iliac artery systems and found 571 patients with abdominal aortic aneurysms and only 11 patients with isolated iliac artery aneurysms, a relative frequency of 1.9%. Later in 1983, McCready et al^10^ reported the frequency of isolated iliac artery aneurysms of 0.9% and provided one of the only anatomical frequency distributions amongst isolated aneurysms of the iliac artery system: 90% of all isolated iliac artery aneurysms affect the common iliac artery solely, whereas <1% affect the EIA solely. Finally, in 1989 Brunkwall et al^5^ performed the largest investigation on isolated iliac artery aneurysms, reporting 13 cases during the 15-year compilation of autopsy and operating records in Malmo Sweden, population 230,000. They found only one isolated EIA aneurysm in that same study.^5^

Regardless of how rare they are, many investigations have demonstrated that isolated iliac artery aneurysm are associated with a high risk of rupture and mortality, with rates of rupture between 14% and 75%.^2^^,^^11^^,^^12^ The high mortality rate is postulated to be due to the lack of inclusion of iliac artery aneurysms in a differential diagnosis of pelvic conditions, partly owing to their rarity. Also, owing to the nature and location of the pathology, they are difficult to detect on physical exam until they are at a size when they are at risk for a morbid rupture.^3^ McCready et al described that 78% of the patients in their study presented asymptomatically with their iliac artery aneurysm, similar to the patient described in this case study.

Few cases have been reported describing isolated aneurysms of the EIA (Table). The first case report in 1952 described a patient who presented symptomatically with abdominal and lower limb pain. Surgical exploration revealed an EIA aneurysm that had ruptured.^13^ The next three case reports published between 1986 and 2009 described iliac artery aneurysms involving the EIA that were found histologically to be due to cystic medial necrosis.^14^, ^15^, ^16^ More recently, in 2019 two cases were presented with a 65-year-old symptomatic patient with left lower limb edema and a 55-year-old symptomatic patient with intermittent left thigh pain associated with paresthesias, both ipsilateral to the isolated EIA aneurysm.^17^^,^^18^ Finally in 2020, there was another case report, similar to Crivello's, describing a symptomatic isolated EIA aneurysm associated with cystic medial necrosis.^19^ In the present report, we have presented the case of a 68-year-old man who was completely asymptomatic, with an incidental finding of an EIA aneurysm. Until the presentation of our case, a search of PubMed found only seven reported cases of isolated EIA aneurysm, none of which were completely asymptomatic or repaired endovascularly.

**Table:** Reported cases of isolated external iliac artery (*EIA*) aneurysm

Author	Age, years	Sex	Size, cm	Year
Priddle et al^13^	29	Female	4.0	1952
Crivello et al^14^	27	Male	Not available	1986
Mohan et al^15^	66	Male	11.0	1997
Kato et al^16^	78	Female	4.0	2009
Van de Luijtgaarden et al^17^	65	Male	3.5	2019
Hussain et al^18^	55	Male	7.0	2019
Chatzantonis et al^19^	51	Male	2.0	2020
Current case	68	Male	2.6	2023

With this case report, we hope to stimulate a discussion on when to intervene on these rare pathologies. Although we have guidelines, based on large sample studies, for when to fix common or internal iliac artery aneurysms, the rarity of isolated EIA aneurysms leaves vascular surgeons without clear directions on when to fix them, especially when they are asymptomatic. Of the different publications reporting sizes of isolated EIA aneurysms, we found that most were repaired between 2 and 4 cm, mostly in men approximately 60 years old. Until clearer, large sample studies are performed, we recommend early repair of these aneurysms (diameter ≥2 cm) owing to the risk of rupture or symptoms seen in prior publications. Additionally, we now have the availability of minimally invasive interventions, with endografts that are able to be surveilled postoperatively with ultrasound examination.

## Funding

None.

## Disclosures

None.
